# Artificial Intelligence Approach to the Monitoring of Respiratory Sounds in Asthmatic Patients

**DOI:** 10.3389/fphys.2021.745635

**Published:** 2021-11-11

**Authors:** Honorata Hafke-Dys, Barbara Kuźnar-Kamińska, Tomasz Grzywalski, Adam Maciaszek, Krzysztof Szarzyński, Jędrzej Kociński

**Affiliations:** ^1^Department of Acoustics, Faculty of Physics, Adam Mickiewicz University in Poznań, Poznań, Poland; ^2^StethoMe Sp. z o.o., Poznań, Poland; ^3^Department of Pulmonology, Allergology and Respiratory Oncology, Poznan University of Medical Sciences, Poznań, Poland

**Keywords:** asthma, monitoring, auscultation, rhonchi, wheezes, stethoscope, breath, phenomena

## Abstract

**Background:** Effective and reliable monitoring of asthma at home is a relevant factor that may reduce the need to consult a doctor in person.

**Aim:** We analyzed the possibility to determine intensities of pathological breath phenomena based on artificial intelligence (AI) analysis of sounds recorded during standard stethoscope auscultation.

**Methods:** The evaluation set comprising 1,043 auscultation examinations (9,319 recordings) was collected from 899 patients. Examinations were assigned to one of four groups: asthma with and without abnormal sounds (AA and AN, respectively), no-asthma with and without abnormal sounds (NA and NN, respectively). Presence of abnormal sounds was evaluated by a panel of 3 physicians that were blinded to the AI predictions. AI was trained on an independent set of 9,847 recordings to determine intensity scores (indexes) of wheezes, rhonchi, fine and coarse crackles and their combinations: continuous phenomena (wheezes + rhonchi) and all phenomena. The pair-comparison of groups of examinations based on Area Under ROC-Curve (AUC) was used to evaluate the performance of each index in discrimination between groups.

**Results:** Best performance in separation between AA and AN was observed with Continuous Phenomena Index (AUC 0.94) while for NN and NA. All Phenomena Index (AUC 0.91) showed the best performance. AA showed slightly higher prevalence of wheezes compared to NA.

**Conclusions:** The results showed a high efficiency of the AI to discriminate between the asthma patients with normal and abnormal sounds, thus this approach has a great potential and can be used to monitor asthma symptoms at home.

## 1. Introduction

Asthma affects 1–18% of the general population in different countries (Global Initiative for Asthma, 2020) and is characterized by chronic airway inflammation causing recurrent attacks of breathlessness, cough and wheezing sounds. All this leads to expiratory airflow limitation. Its symptoms differ in intensity and severity over time. The variations may be triggered by different factors such as exercise, allergen or irritant exposure, change in weather, or respiratory infections (Global Initiative for Asthma, 2020). In recent decades asthma prevalence has increased worldwide, mainly due to environmental and lifestyle risk factors, particularly in children. Nunes et al. (2017) estimates the mean cost per patient per annum to be $USD 1900 in Europe and $USD 3100 in the USA. Uncontrolled asthma leads to much more significant increase of treatment cost (Sullivan et al., 2014).

In order to reduce social and personal impact of asthma on patients, the key is to achieve good symptom control mainly due to monitoring. Such control minimizes risk of asthma-related mortality, exacerbations, persistent airflow limitation and adverse events of treatment (Global Initiative for Asthma, 2020). This allows asthmatic subjects to maintain normal activity levels resulting in reduction of the overall impact of the disease in society.

The Global Initiative for Asthma (GINA) questionnaire (Global Initiative for Asthma, 2020) defines wheezing (that includes two continuous sound phenomena: wheezes and rhonchi) as a key asthma symptom that must be monitored since it is a typical indicator of obstructed airflow (Pasterkamp, 2017) and is the most common and specific symptom aligned with asthma (Daines et al., 2019; Global Initiative for Asthma, 2020). Other commonly recognized adventitious lung sounds are fine and coarse crackles, which are typically associated with other medical conditions e.g., pneumonia. However, for asthma patients, it may be very difficult, if not impossible, to assess the level of wheezes in the respiratory tract at home. This is in particular due to the difficulty in distinguishing wheezing from crackling, especially coarse crackles which are often mistaken for rhonchi, and vice versa. This task is not trivial even for skilled medical professionals (Pasterkamp, 2017; Hafke-Dys et al., 2019). Finally, the interpretation of wheezes differs based on the person who observes them, the environmental and the cultural context (Global Initiative for Asthma, 2020). Therefore, a quantitative metric that defines an objective level of observed wheezes in asthmatic patients could enhance asthma monitoring.

Usually asthma patients have regular visits every 3–6 months so a physician must rely on their subjective opinion or opinion of parents in the case of children. As suggested by Carroll et al. (2011), patient's opinions significantly differ from the physician's assessment. This can lead to either poor treatment of asthma or overdiagnosis of asthma or exacerbations.

The recent epidemic, moreover, highlights new risks and obstacles to healthcare that may become more common in the future. One of the most important findings in this context is that telemedicine has become a recognized tool of communication between patient and physician (Rasmussen et al., 2005; Mann et al., 2020; Vafea et al., 2020).

Some scientific and commercial solutions related to remote medical care of respiratory conditions have begun to be developed in the last few years. Multiple publications have focused on wheeze detection using various signal processing techniques and machine learning (Pramono et al., 2019). Lin and Lin (2016) used frequency cepstral coefficients (MFCCs) in combination with a gaussian mixture model (GMM) on recordings from 9 asthmatic and 9 healthy patients to detect the presence of wheezes resulting in a sensitivity of 88.1% and specificity of 99.5%. Riella et al. (2009) used a multi-layer artificial network on a dataset of 112 recordings and achieved 84.82% accuracy in wheeze detection, while Lin et al. (2015) used a back-propagation neural network to detect wheezes in a set of 32 asthmatic and 26 healthy patients resulting in sensitivity of 94.6% and specificity of 100%. Other research focused on asthma detection or measuring asthma severity. For example, Shaharum et al. (2016) used a k-nearest neighbors (KNN) algorithm to classify the asthma severity on three levels (mild, moderate, and severe) and achieved a 97.5% accuracy based on wheeze detection in recorded signals. The cited works yielded promising results, however they were tested on limited datasets and groups of patients and their performance might not scale to larger populations. Comparison of datasets and reported results are shown in Table 1.

**Table 1:** Comparison of datasets used in other papers with best classification results reported (if available).

**Article**	**Dataset**	**Sensitivity**	**Specificity**	**Accuracy**
Lin and Lin (2016)	18 adult patients (9 asthma, 9 healthy)	0.881	0.995	N/A
Riella et al. (2009)	112 respiratory cycle recordings (40 wheezes, 72 without wheezes)	0.861	0.825	0.848
Lin et al. (2015)	58 adults (32 asthma, 26 healthy)	0.946	1.000	N/A
Shaharum et al. (2016)	30 mild asthma, 25 moderate asthma and 35 severe asthma samples	N/A	N/A	0.975
Ra et al. (2016)	63 wheezing sounds, 40 normal sounds	1.000	0.950	0.980

Adejumo and Shaw (2018) state that electronic monitoring devices (EMD) hold promise and with further technological development, carefully considered study design, and better understanding of patient barriers such devices could lead to lower morbidity and mortality in asthmatic patients. Multiple EMDs were created and tested. Satat et al. (2016) presented an EMD for home monitoring of asthma in children using multiple stethoscopes while Koehler et al. (2018) showed an automated respiratory sound monitoring device, which records respiratory sounds continuously by three small bioacoustical sensors attached to the trachea and to the back of the patient. Furthermore, Ra et al. (2016) introduced a system for daily asthma monitoring using an array of microphones, a smartphone and a cloud service to have sensitivity of 98.4% and specificity of 95% in detecting wheeze sounds on a set of 103 recordings. Such EMDs are very accurate but they require non-standard or sophisticated hardware making the process hard or unfeasible in real-life scenarios.

In this paper, we propose a novel approach to acoustic data analysis that may essentially help in remote control and management of asthma patients. This is based not just on wheeze/rhonchi detection, but additionally on a unique approach to quantitative measurement of the intensity of these sounds. The main aim of our research is to assess whether a quantitative measure of respiratory pathological phenomena intensity computed by an AI algorithm can be used for asthma monitoring.

## 2. Materials and Methods

### 2.1. Lung Sounds Database

In this study, a proprietary large-scale database of lung sounds was used. The database was built with signed consent from the patients or the parents of under-age patients and was approved by a bioethical commission. Database recordings were gathered between November 2017 and January 2021 by medical doctors during standard auscultation procedures in their daily practice. Each examination included up to 12 recordings registered in different locations on the thorax. From this database two samples were taken.

First sample comprising 9,847 recordings from 1,120 real-life examinations was used to develop the proposed AI solution. Second sample was used for validation. It consisted of all examinations of patients that were not included in the first sample and were not diagnosed with asthma with comorbidities. Recordings in the validation sample were further processed to check their quality. A trained acoustician with experience in analysis of lung sounds assessed each recording and if he determined that no breathing cycle could be heard or too much background noise was present, the recording was rejected. Eventually all examinations with less than four valid, good quality recordings were removed.

The final dataset used for validation included 1,043 examinations gathered from 899 patients. This corresponded to 11,000 unique recordings, of which 9,319 were good quality recordings. The vast majority of examinations were performed on unique patients, but in some cases one patient could be examined up to three times. Two thousand eight hundred and thirty-one signals were recorded with Littmann 3200 and 8169 with StethoMe stethoscopes. The lengths of recordings ranged from 2.6 to 61.6 s, averaging to 14.6 s. A total of 33 medical doctors contributed to this dataset, either in the form of sound recordings, labeling or verification.

In Supplementary Materials, one can find the raw data that were used to calculate results of this paper. For research purposes we encourage everyone to send us the auscultatory recordings, the algorithm will analyze the data and we send the results back free of charge.

### 2.2. Proposed AI Solution

The output data of our AI algorithm is described with the help of seven indexes describing pathological breath sound intensities. Four base indexes represent the intensities of the four basic types of adventitious breath sounds present in an auscultation examination, namely:

- Wheezes Index
- Rhonchi Index
- Fine Crackles Index
- Coarse Crackles Index

These sounds are identified using nomenclature proposed by Pasterkamp (wheezes, rhonchi, fine crackles, coarse crackles) (Pasterkamp et al., 2015; Grzywalski et al., 2019), which is recommended by the European Respiratory Society, the International Lung Sounds Association, and the American Thoracic Society. In addition to these indexes, we also estimate the following three joint indices for the following combinations of sounds:

- Continuous Phenomena Index (wheezes and rhonchi)
- Transient Phenomena Index (fine and coarse crackles)
- Overall Index (all phenomena).

The development of the proposed AI solution started with the manual tagging of the first data sample (the development dataset). This process was split into two stages. First, medical professionals provided two sets of labels: a single label for the whole auscultation examination and the four base labels for each examination recording. Later we will refer to these as ground truth examination-level labels and recording-level labels, respectively. The examination-level label represented the patient's overall lung health state at the time of auscultation described on a three-point scale:

- label 0: no or negligible abnormal sounds of any type
- label 1: some moderate abnormalities in sounds present
- label 2: large numbers of adventitious pathological sounds

**Figure 1:**
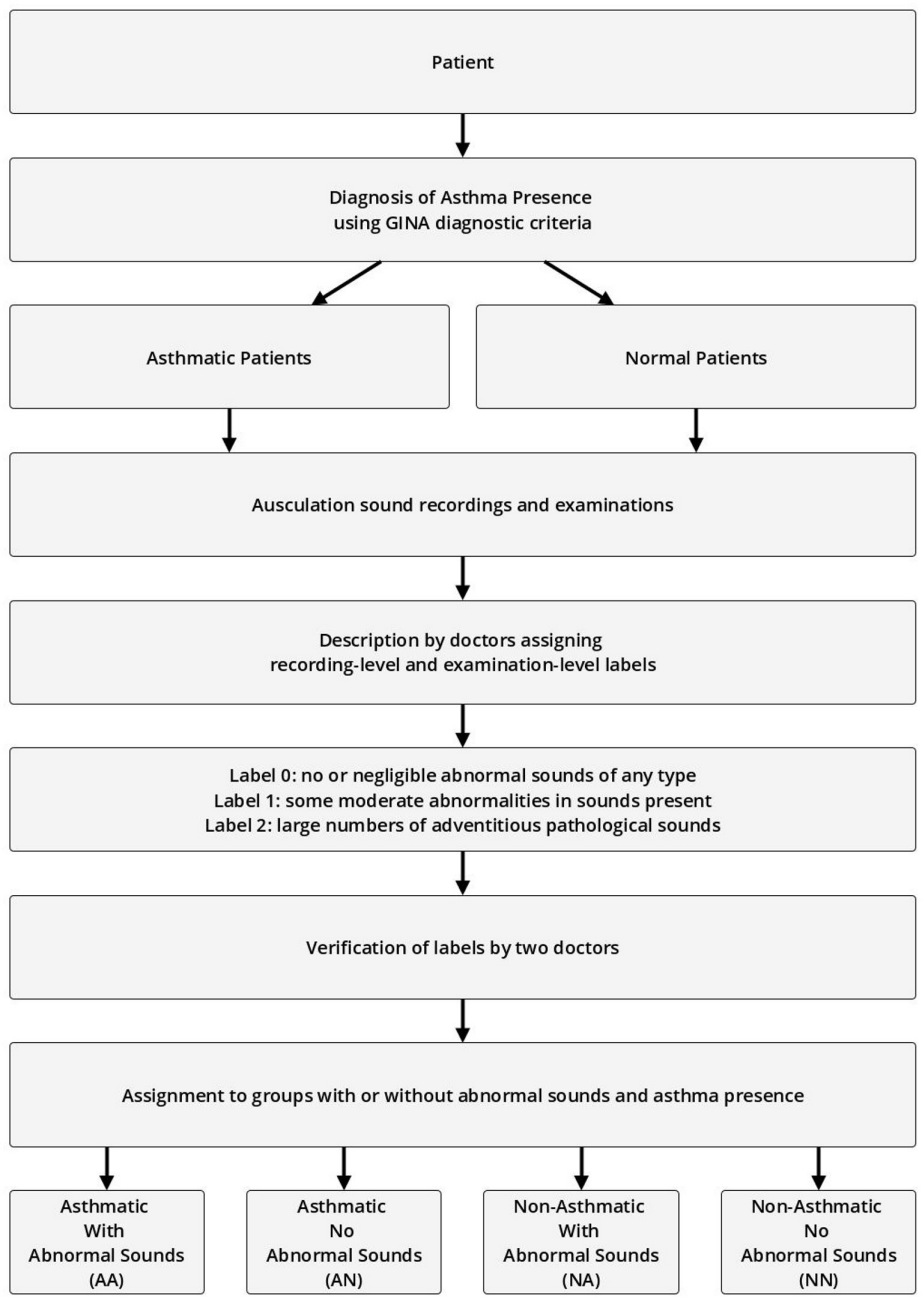
Process of assigning patients, examinations, and recordings to groups based on presence of abnormal sounds and asthma diagnosis.

The recording-level labels describe the presence of the four basic pathological breath phenomena in each recording on an analogous three-point scale (no/negligible, moderate, high intensity). Each label was approved by at least two other medical professionals before it was accepted for further use. The process of labeling and assigning of patients to specific experiment groups is shown in Figure 1.

In the second stage, based on examination and recording-level labels, professional and trained acousticians prepared frame-level annotations of breath sounds. Every sound present in each recording was identified by providing a beginning and end timestamp (i.e., start and end time of the phenomenon) and a sound type label: wheeze, rhonchi, fine crackle, coarse crackle, inspiration, expiration, or noise. Since these sounds often co-occur, more than one annotation could have been present at any given audio frame of a recording. At this stage, the protocol required a consensus between at least two acousticians for the annotation to be accepted.

**Figure 2:**
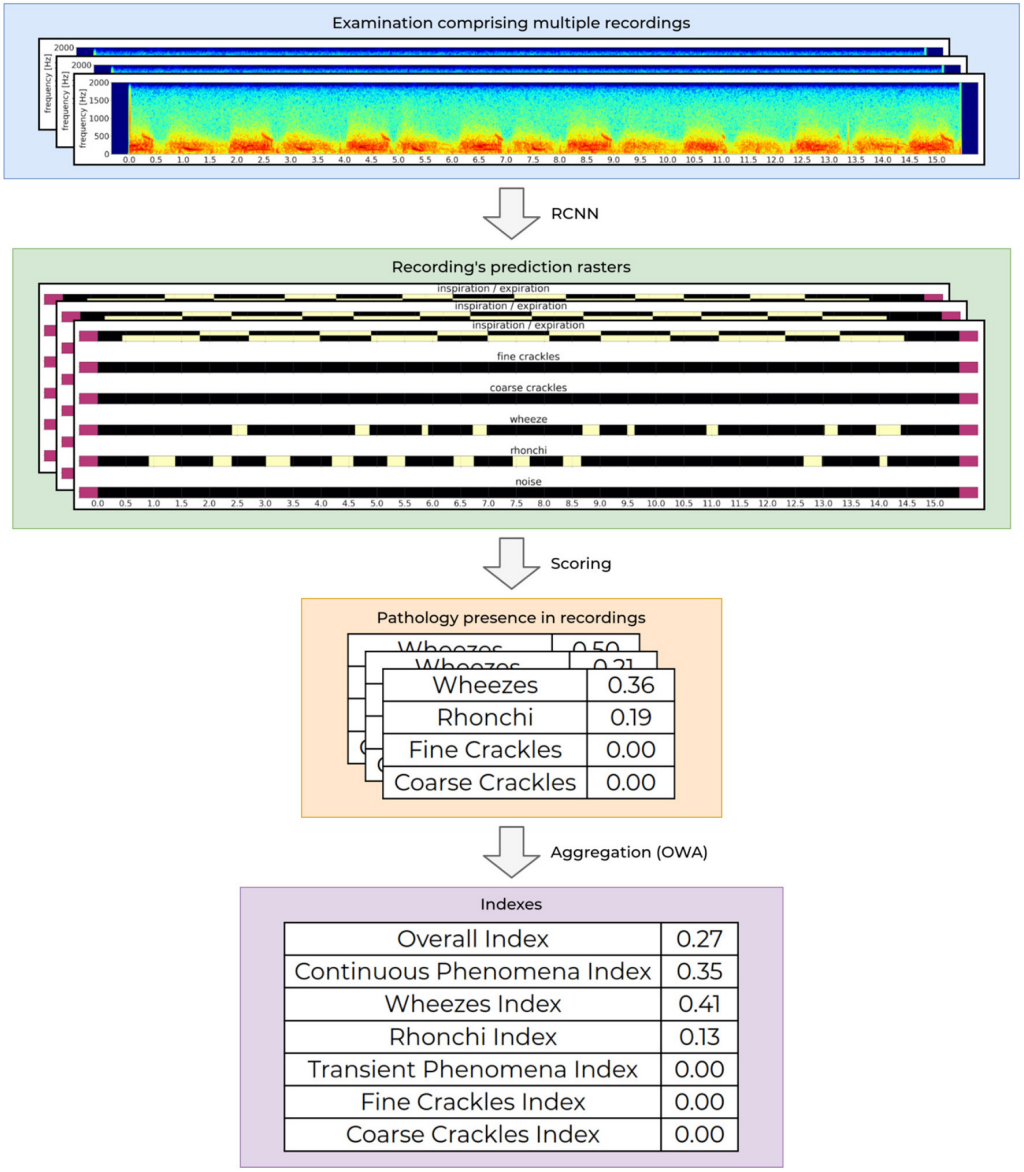
Process of determining the indexes values. The processing starts with an audio recording acquisition and analysis, through aggregation of the analysis from one recording to calculation of the final indexes values for the entire examination.

At the core of our AI module is a recurrent-convolutional neural network (RCNN), very similar to the one presented by Grzywalski et al. (2019) trained on the frame-level annotations of breath sounds. At the input the network accepts a single examination recording and outputs a matrix called the prediction raster. It contains information about the presence of a particular breathing sound at a given time-point of the recording. Figure 2 shows an example of an input examination recording (first row) and matching RCNN prediction raster (second row).

The first layer of the network consists of a single convolution layer that processes an audio sample (array of audio samples) and approximates a STFT operation on the audio signal, then a series of eight 2-dimensional convolution layers with batch normalization are used to extract valuable parts of the spectrogram. Finally a series of three bidirectional gated recurrent unit (GRU) layers described by Cho et al. (2014) are used to analyse the extracted features in time-domain. The whole model contains approximately 7.4 million trainable parameters and trains for about 24 h on a modern GPU. The dataset used to train the RCNN was separate and did not contain recordings from patients that were used to validate the proposed AI solution.

To analyse intensities of breathing sounds in a recording, each raster generated by the network is processed further to calculate the overall presence of a given pathological sound. This is based on an analysis of correlation of detected phenomena, detected breathing cycle and detected noise in the recording. The obtained values are scaled to fit to the ground truth recording-level labels. This results in a set of four values, one for each abnormal breathing sound (wheezes, rhonchi, fine, and coarse crackles) ranging from 0.0 to 1.0. Low values mean that the given recording contains no or negligible pathological sounds of a particular kind, while high values correspond to a high prevalence of a given pathological sound in the recording. This functionality is already publicly available as a CE2274 certified medical device named StethoMe AI.

Intensities of pathological breath sounds from individual recordings are aggregated using an ordered weighted averaging aggregation operator (OWA) and scaled to fit the ground truth examination-level labels to yield a single floating point examination index. The OWA weights that are used to calculate the respiratory indexes is based on continuous scale by non-linear discretization based on the empirical data from tags. The index value ranges from 0.0 when no or very little abnormal sounds are present in the examination to 1.0 for patients with a high number of pathological sounds present. The whole process of determining the indexes values is depicted in Figure 2.

OWA inputs can be chosen in multiple ways. For example the index can take into account multiple combinations of pathological sound types, in particular all types can be used (wheezes, rhonchi, fine, and coarse crackles), single pathology types (e.g., just wheezes) or various combinations of thereof (e.g., continuous phenomena—wheezes and rhonchi). In this research seven indexes were considered as described at the beginning of this section.

### 2.3. Study Design

The validation examinations (second data sample) were divided into four groups:

- Asthmatic With Abnormal Sounds (AA)
- Asthmatic No Abnormal Sounds (AN)
- Non-Asthmatic With Abnormal Sounds (NA)
- Non-Asthmatic No Abnormal Sounds (NN)

**Figure 3:**
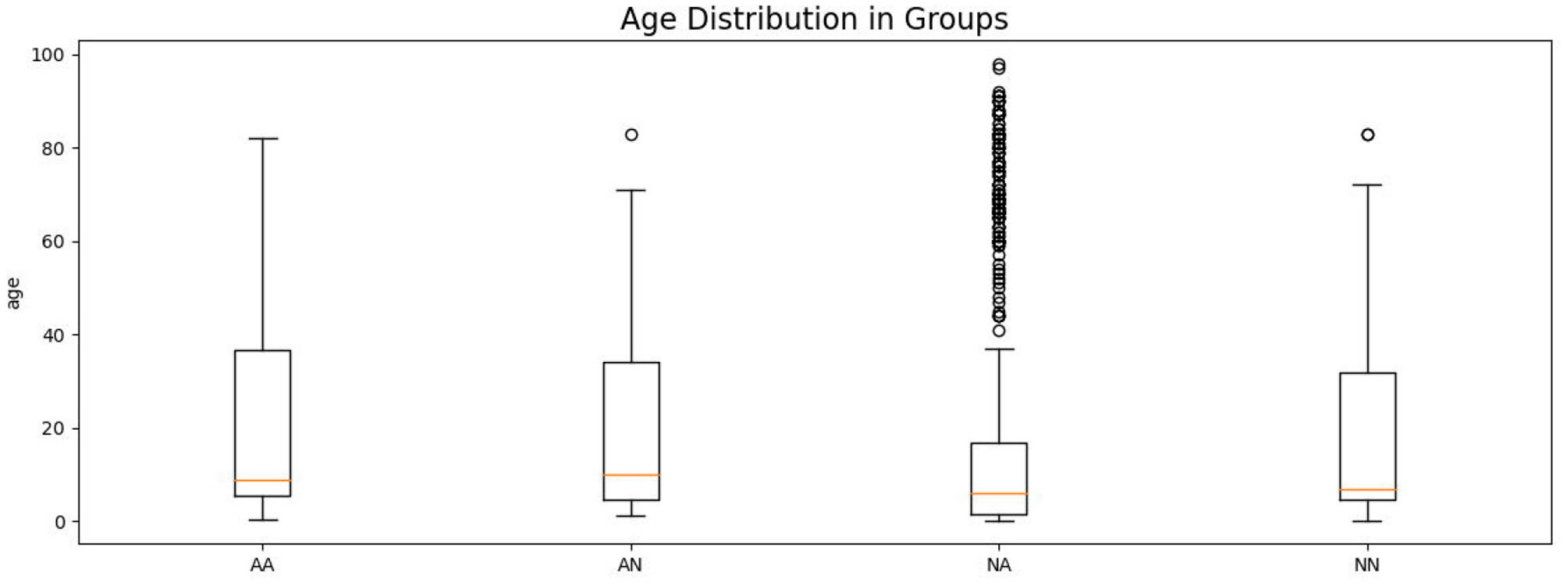
Age distribution of patients in each comparison group shown as a box plot. The box extends from the lower to upper quartile values of the data, with a line at the median. The whiskers extend from the box to show the range of the data. Flyer points are those past the end of the whiskers.

Asthmatic patients are patients diagnosed with asthma by an experienced medical professional using diagnostic criteria defined by Global Initiative for Asthma (GINA) (Global Initiative for Asthma, 2020). Since there is no standardized (international or local) way of detecting and describing lung sounds, we developed our own protocol to classify each examination as either No Abnormal Sounds or With Abnormal Sounds. Abnormal sounds tagged as present means that (a) in the data description stage a medical professional identified adventitious sounds (wheezes, rhonchi, fine, or coarse crackles) in the stethoscope examination, and (b) this assessment was positively verified by at least two other medical professionals. The medical personnel involved in this process were blinded to the AI predictions. Details about composition of the validation dataset split into the four groups of patients is presented in Table 2 and the age distribution of patients is presented in Figure 3. The dataset consisted mostly of young adult patients (mean age of all patients was 19 with standard deviation of 25.2) however it included patients covering all age groups.

**Table 2:** Validation dataset.

**Group of examinations**	**AA**	**AN**	**NA**	**NN**
Number of examinations	62	57	761	163
-Including male	22	32	408	89
-Including female	40	25	353	74
Number of recordings	721	457	7,988	1,834
-Incl. good quality recordings	661	386	6,781	1,491
Patient age				
-Mean	19.4	21.8	18.7	19.1
-Std	20.7	22.3	26.8	25.2

High imbalance in the number of auscultation examinations between groups, especially the high number of NA examinations results from the fact that in the data gathering step no particular exclusion or inclusion criteria were used (except for exclusion of asthmatic patients with comorbidities). Out of a total of 823 patients' examinations with adventitious lungs sounds, only 62 were acquired from patients diagnosed with asthma, which reflected the natural proportion in the population of people that were seeking medical help from doctors that contributed to the lung sounds database.

The seven indexes provided by the proposed AI solution were then evaluated in terms of their ability to differentiate two groups of examination results. The following comparisons were considered:

- AA vs. AN
- AN vs. NN
- AA vs. NA
- NA vs. NN

We decided not to perform cross comparisons (AA vs. NN and AN vs. NA) as we find them less meaningful, since one cannot deduce which factor is responsible for any difference that may appear in results.

### 2.4. Methods

We calculated the Area under ROC-curve (AUC) (Bradley, 1997) as a measure of separation that can be achieved between groups within each considered pair using a given index. AUC ranges from 0.0 to 1.0. High AUC values (close to 1.0) mean that the two groups can be easily separated using the index, whereas values around 0.5 correspond to no such distinguishing capability. Low values of AUC (close to 0.0) also indicate good separation, but with reversed correlation, namely high index values are associated with high probability that the examination belongs to the second group of patients, not the first.

## 3. Results

**Figure 4:**
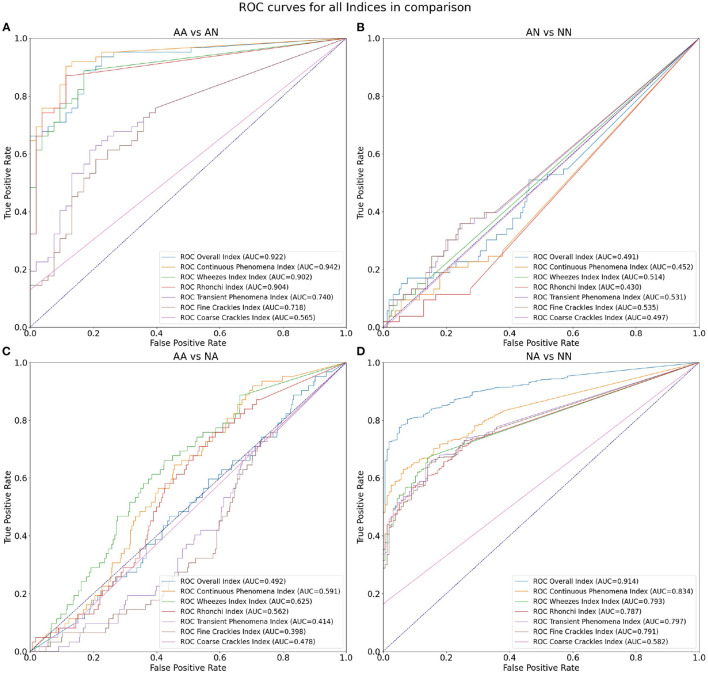
ROC curves for all indices in comparison: **(A)** differentiation of AA and AN groups, **(B)** AN and NN groups, **(C)** AA and NA groups, and **(D)** NA and NN groups.

As the first outcome of our experiment, in Table 3 we present the statistics regarding mean and standard deviation of index values within each of the four groups of examinations. Next, in Figure 4 we present the ROC curves for all indexes and all considered pairs of groups of examinations. Finally in Table 4, we summarize the obtained ROC values that illustrate how well the groups within each pair may be separated using each index. Finally we calculated classification metrics (sensitivity, specificity, and accuracy) for all comparisons and indexes. To obtain the classification metrics for each comparison group and index type we selected an optimal threshold that balances the values of sensitivity and specificity. Using this threshold we calculated sensitivity, specificity, and accuracy. The results for comparison groups and best indexes are presented in Table 5.

**Table 3:** Mean and standard deviation of each index value within each group of examinations.

**Group of examinations**	**AA**	**AN**	**NA**	**NN**
Overall index	0.37 ± 0.33	0.02 ± 0.04	0.39 ± 0.35	0.02 ± 0.03
Continuous phenomena index	0.30 ± 0.29	0.01 ± 0.02	0.26 ± 0.32	0.01 ± 0.02
Wheezes index	0.14 ± 0.16	0.01 ± 0.01	0.11 ± 0.18	0.00 ± 0.01
Rhonchi index	0.17 ± 0.23	0.01 ± 0.02	0.17 ± 0.24	0.01 ± 0.02
Transient phenomena index	0.08 ± 0.12	0.01 ± 0.03	0.16 ± 0.23	0.01 ± 0.02
Fine crackles index	0.06 ± 0.12	0.01 ± 0.03	0.13 ± 0.19	0.01 ± 0.02
Coarse crackles index	0.02 ± 0.06	0.00 ± 0.00	0.03 ± 0.12	0.00 ± 0.00

**Table 4:** AUCs for all index values and four considered comparisons.

**Comparison**	**AA vs. AN**	**AN vs. NN**	**AA vs. NA**	**NA vs. NN**
Overall index	0.922	0.491	0.492	**0.914**
Continuous phenomena index	**0.942**	0.452	0.591	0.834
Wheezes index	0.902	0.514	**0.625**	0.793
Rhonchi index	0.904	0.430	0.562	0.787
Transient phenomena index	0.740	0.531	0.414	0.797
Fine crackles index	0.718	**0.535**	0.398	0.791
Coarse crackles index	0.565	0.497	0.478	0.582

*The highest ROC values for each comparison are marked with bold font*.

**Table 5:** Classification results for all comparisons and best index in each group.

**Comparison group**	**Index type**	**Sensitivity**	**Specificity**	**Accuracy**
AA vs. AN	Continuous phenomena	0.887	0.887	0.887
AN vs. NN	Overall	0.321	0.763	0.651
AA vs. NA	Wheezes	0.581	0.639	0.635
NA vs. NN	Overall	0.809	0.891	0.823

## 4. Discussion

Our results show that the highest mean value of Overall Index is in the NA group (0.39) followed by AA (0.37) (Table 3). The two groups with no abnormal sounds have much smaller values of mean Overall Index (0.02 in both AN and NN groups). This is perfectly justifiable as patients with abnormal sounds presence assignment are expected to have adventitious lung sounds. It shows that the Overall Index may be used for determining whether a subject has pathological phenomena sounds in the lungs or not. The Overall Index mean value in the NA group may possibly be higher than in the AA group due to the presence of patients with more severe medical conditions than asthma, i.e., pneumonia.

Comparing both asthmatic groups (abnormal sounds presence vs. no abnormal sounds presence) it is evident that the examinations of the abnormal sounds presence group contain more pathological lung phenomena of all kinds. This pair of groups is characterized by the maximal AUC value attained among all studied pairs and indices—0.942 for the Continuous Phenomena index (Table 4). The degree of separation using Overall, Continuous Phenomena, Wheezes, and Rhonchi Indices is high, meaning that usually when asthmatic patients have pathological lungs sounds, these are continuous phenomena—wheezes or rhonchi, which is in line with the literature (Global Initiative for Asthma, 2020). It shows that these indices are good measure candidates to monitor asthma patients' auscultatory changes.

When comparing AA and NA groups more closely, it is important to note that in the asthmatic group there are more continuous phenomena, especially wheezes. Analyzing Table 3 one can see that the Continuous Phenomena Index is on average higher by 0.04 and Wheezes Index by 0.03 in the AA group, whereas the Rhonchi Index averages are exactly at the same level of 0.17 in both groups. The Transient Phenomena Index, Fine Crackles Index, and Coarse Crackles Index values averages are higher in the non-asthmatic group. This is in line with the state of the art in the asthma domain as transient phenomena are less representative of asthma than continuous phenomena (Global Initiative for Asthma, 2020). Analyzing AUC values of the AA vs. NA groups it is noticeable that the Index that separates the groups best is the Wheezes Index. Its value is 0.625 (Table 4) so the separation is far from perfect. This is due to the fact that in the NA group there are also patients with pathological wheeze sounds in the lungs so it is not possible to distinguish between the groups solely using a pathological lung sound index.

The degree of separation between NA vs. NN (Table 4) as measured by AUC is also very significant, reaching 0.914 with Overall Index while the Continuous Phenomena Index (0.834) shows no clear advantage over Transient Phenomena Index (0.797) and all Indices except the Coarse Crackles Index attain comparable values. This could mean that all phenomena, except coarse crackles which are overall the least frequent in the population, occur with a similar frequency in non-asthmatic groups. This is also in line with the literature (Global Initiative for Asthma, 2020).

As can be seen in Table 4 the smallest degree of separation is observed in the AN vs. NN comparison. These groups contain examinations of patients with no pathological sounds and therefore cannot be easily separated using the indices.

When comparing the classification metrics in Table 5 we can confirm previous findings. The Overall Index performs very good when used for distinguishing between patients with and without abnormal sounds in the asthmatic (sensitivity of 0.919, specificity of 0.774, and accuracy 0.852) and non-asthmatic groups (sensitivity of 0.938, specificity of 0.506, and accuracy of 0.863). In case of the asthmatic patients we also see that the Continuous Phenomena Index (including wheezes and rhonchi) achieves good results in classifying presence of abnormal sounds with sensitivity of 0.887, specificity of 0.887, and accuracy of 0.887.

In groups with smallest degree of separation, namely AN vs. NN and AA vs. NA we see that highest specificity and accuracy are achieved using the Coarse Crackles Index (1.000 and 0.746 for AN vs. NN, and 0.834 and 0.779 for AA vs. NA, respectively).

Comparing directly classification results of our proposed solution with ones discussed in the introduction of this paper shows at first our solution is slightly inferior to them. However, it is important to note that our research is based on significantly bigger dataset of recordings. For example, the biggest discussed dataset was present in Lin and Lin (2016)—58 adults (32 with asthma, 26 healthy) while our dataset contains recordings of 899 patients.

## 5. Conclusions

The results shown in the paper suggest that the Artificial Intelligence approach proposed here may be a very good tool for monitoring the respiratory system state of asthma patients. It can distinguish between stable states and exacerbations leading to an automatic monitoring of asthma. The results of this paper are in line with the findings by Kevat et al. (2020) who confirmed the high efficiency of a general automatic AI approach to the detection of wheezes, rhonchi, coarse, and fine crackles. The present approach makes use of those findings and goes one step further to provide the tool for monitoring the most common acoustic symptoms of asthma.

It is worth noting that detection of additional respiratory sounds alone is not sufficient to diagnose asthma, but it is information that can be used to effectively monitor the disease. Wheezing and rhonchi are signs of obstruction in the respiratory system, which is present during an asthma exacerbation event. The appearance of these additional phenomena is a clear indication to the patient and doctor that appropriate action must be taken—either to take bronchodilator or to modify the long-term treatment plan. In controlled asthma, these sounds should not be permanently present in the respiratory system. It can be found in the literature that most of the asthma exacerbation cases are characterized by appearance of many abnormalities and their intensity (Global Initiative for Asthma, 2020), thus discrimination seems possible. Furthermore, some severe asthma exacerbation result in a so-called “silent chest,” when there are no abnormal sounds but the respiratory tract is almost completely closed. Nevertheless, in such cases other symptoms, making it possible to react. One must also bear in mind that the proposed solutions is not capable of discrimination between some groups (i.e., AN vs. NN and AA vs. NA). It means that its full potential can be utilized once one is diagnosed and wants to monitor the asthma state.

Moreover, our AI based solution is, in principle, tailored for remote use applications and does not necessarily require continuous and direct oversight by doctors while the high quality results may be easily transferred on demand to and used by specialists for further analysis and treatment decisions. Indeed, the results generated by the proposed system are accurate and have the potential to effectively support examination and remote monitoring whenever in-person examination is unfeasible. Furthermore, this easy and fast way of asthma state monitoring may lead to much better and more precise analysis of the patient's state leading to treatment optimization. Finally, self monitoring of asthma by the patient may also help raise disease awareness and render this chronic disease less burdensome. It thus holds the promise of more flexible and reliable health care.

To conclude, monitoring and treatment adjustments tailored to patients needs are essential to achieve controlled asthma and are therefore a fundamental part of comprehensive asthma management (van den Wijngaart et al., 2016).

## Data Availability Statement

The raw data supporting the conclusions of this article will be made available by the authors, without undue reservation.

## Ethics Statement

The studies involving human participants were reviewed and approved by Poznan University of Medical Sciences. Written informed consent to participate in this study was provided by the participants' legal guardian/next of kin.

## Author Contributions

HH-D conceptualized and designed the study, drafted the initial manuscript, and finally approved the version of the manuscript. BK-K supervised the medical aspects of the study, reviewed and revised the manuscript, and finally approved the version of the manuscript. TG conceptualized and developed the AI part of the study, analyzed and described the results of this analysis. AM and KS developed the AI part of the study, drafted the initial manuscript, and prepared all the figures. JK reviewed and revised the manuscript and analyzed the data, drafted the initial version of the manuscript, and critically reviewed the manuscript for important intellectual content. All authors approved the final manuscript as submitted and agreed to be accountable for all aspects of the work.

## Funding

This work was supported by the grant no. POIR.01.01.01-00-0648/20 from The National Centre for Research and Development.

## Conflict of Interest

Hereby we state that HH-D and JK are employed both in Adam Mickiewicz University in Poznań and StethoMe Company and are the shareholders of StethoMe. TG, AM, and KS are employed in StethoMe Company and BK-K is employed in Department of Pulmonology, Allergology and Respiratory Oncology, Poznan University of Medical Sciences and has nothing to disclose. All authors were paid for their work. This does not alter our adherence to Frontiers policies on sharing data and materials, and the results were obtained with all the ethical standards and objectivity.

## Publisher's Note

All claims expressed in this article are solely those of the authors and do not necessarily represent those of their affiliated organizations, or those of the publisher, the editors and the reviewers. Any product that may be evaluated in this article, or claim that may be made by its manufacturer, is not guaranteed or endorsed by the publisher.
